# Self-reported bruxism mirrors anxiety and stress in adults

**DOI:** 10.4317/medoral.18232

**Published:** 2012-08-28

**Authors:** Jari Ahlberg, Frank Lobbezoo, Kristiina Ahlberg, Daniele Manfredini, Christer Hublin, Juha Sinisalo, Mauno Könönen, Aslak Savolainen

**Affiliations:** 1 Institute of Dentistry, University of Helsinki, Helsinki, Finland; 2 Department of Oral Kinesiology, Academic Centre for Dentistry Amsterdam (ACTA), Research Institute MOVE, University of Amsterdam and VU University Amsterdam, Amsterdam, The Netherlands; 3 Department of Maxillofacial Surgery, University of Padova, Italy; 4 Finnish Institute of Occupational Health, Helsinki, Finland; 5 Helsinki University Central Hospital, Helsinki, Finland; 6 Finnish Broadcasting Company, Helsinki, Finland

## Abstract

Objectives: The aims were to analyze whether the levels of self-reported bruxism and anxiety associate among otherwise healthy subjects, and to investigate the independent effects of anxiety and stress experience on the probability of self-reported bruxism. Study Design: As part of a study on irregular shift work, a questionnaire was mailed to all employees of the Finnish Broadcasting Company with irregular shift work (number of subjects: n=750) and to an equal number of randomly selected employees in the same company with regular eight-hour daytime work. Results: The response rates were 82.3% (56.6 % men) and 34.3 % (46.7 % men), respectively. Among the 874 respondents, those aware of more frequent bruxism reported significantly more severe anxiety (p<0.001). Adjusted by age and gender, frequent bruxers were more than two times more likely to report severe stress (odds ratio 2.5; 95% confidence interval 1.5-4.2) and anxiety (odds ratio 2.2; 95% confidence interval 1.3-3.6) than non-or-mild bruxers. Conclusions: Present findings suggest that self-reported bruxism and psychological states such as anxiety or stress may be related in working age subjects.

** Key words:**Bruxism, self-report, anxiety, stress, adult.

## Introduction

In psychologically healthy adult populations (namely, persons without a severe anxiety disorder or other major psychological problems) the relationship of anxious mood and bruxism has remained inconsistent (1-10). However, it seems that especially transient anxious reaction to stressful event may relate to self-reported bruxism.

Sleep bruxism has been shown to be part of complex arousal response of the central nervous system, which occurs during changes in sleep depth and is accompanied by, among others, body movements, increased heart rate, respiratory changes, and muscle activities (11-14). Since sleep problems, frequent awakenings in particular, was found common in our previous study (15), it is possible that underlying anxiety and stress may exacerbate bruxism along with more frequent arousals during sleep. Whether this applies to self-reported bruxism remains unclear.

Recent studies performed on multiprofessional media personnel, who could be considered as under sustained pressure at work due to irregular shifts, intense on-going technological changes, deadlines, and demands involved in direct broadcasting, suggest that subjectively conceptualized awareness of bruxism (i.e., self-reported grinding or clenching, during sleep or while awake) may reveal, among others, perceived stress (16) and dissatisfaction with one’s work shift schedule (17). However, those previous studies have neither controlled for anxiety nor assessed the relationship of anxiety and severity of bruxism. Thus, the aim of the present study was to further investigate whether anxiety and stress experiences, both measured with validity tested me-thods, associated with levels of self-reported bruxism.

## Material and Methods

As part of comprehensive study on the effects of irregular shift work on health and work performance, a questionnaire was mailed in 2003 to all employees of the Finnish Broadcasting Company with irregular shift work (number of subjects: n=750; 57.0 % men) and to an equal number of randomly selected employees in the same company with regular eight-hour daytime work (42.4 % men) (8-9). The work duties of the media personnel included journalism, broadcasting, programme production, technical support, and administration. The overall response rate was 58.3% (n=874; 53.7% men). The response rate in the irregular shift work group was 82.3% (56.6% men) and in the regular daytime work group 34.3% (46.7% men). The mean age of males in shift work was 45.0 (standard deviation: SD 10.6) years and of females 42.6 (SD 10.7) years. The corresponding figures for daytime workers were 47.4 (SD 9.7) and 45.5 (SD 10.1) years, respectively. Notwithstanding the uneven response rates, the invited subjects and respondents in the shift work and day work groups were similar as regards gender and age, which suggests that the day work group may also be representative. However, as the present study was not any longer targeted to examine the work group aspect, all respondents were included to ensure more power. Ethics clearance was obtained from the Committee of Occupational Health, Helsinki and Uusimaa Hospital District, Helsinki, Finland.

Based on the survey data described above, the following variables were included in the present study (n=874):

a) Demographic data: gender, age, work type.

b) Bruxism: self-assessed awareness and frequency of tooth clenching or grinding with a five-point scale (never, seldom, sometimes, often, and continually). Subjects reporting bruxism ‘often’ or ‘continually’ were categorized as ‘frequent bruxers’, those reporting bruxism ‘sometimes’ as ‘moderate bruxers’, and the rest as ‘non-or-mild bruxers’. ‘Bruxism’ is used as general term throughout the manuscript, unless stated otherwise.

c) Anxiety: the 10-item subscale of the Symptom Checklist-90 (SCL-90-R) (18). The five-point scale ranged from ‘not at all’ to ‘very much’.

d) Stress (Occupational Stress Questionnaire) (19): level of perceived stress, measured and classified with a five-point scale as follows: ‘Stress means the situation when a person feels tense, restless, nervous or anxious, or is unable to sleep because his/her mind is troubled all the time. Do you feel that kind of stress these days?’ (not at all, only a little, to some extent, rather much, very much). Those currently reporting stress ‘rather much’ or ‘very much’ were categorized to have ‘severe stress’.

-Statistical methods

The data were cross-tabulated and the χ2 test was used to study associations between categorical variables. The Jonckheer-Terpstra test (a non-parametric trend test) was used to assess whether the severity of bruxism and anxiety were correlated. Multinomial logistic regression model was used to analyze the independent effects of the psychological variables on the probability of moderate and frequent bruxism. The reference category was non-or-mild bru-xism. Variables for the multivariate analyses were categorized as follows: age (in years), gender (male = 0, female = 1), irregular shift work (no = 0, yes = 1), severe stress (no = 0, yes = 1), anxiety (score above the Finnish norm for community subjects = 1, else = 0) (18). The multivariate model was also tested excluding the work group variable, which did not markedly change the effects of the other independent variables. Thus, despite the uneven response rates, the work group variable was not considered to cause confounding interaction in the model and it was used as an adjust variable. Odds ratios (OR) and their corresponding 95% confidence intervals (CI) were calculated.

## Results

 Table 1 shows the crude associations between the study variables and the severity of self-reported bruxism; perceived anxiety and severe stress were both found significantly associated with more frequent bruxism (p< 0.001).

**Table 1:**
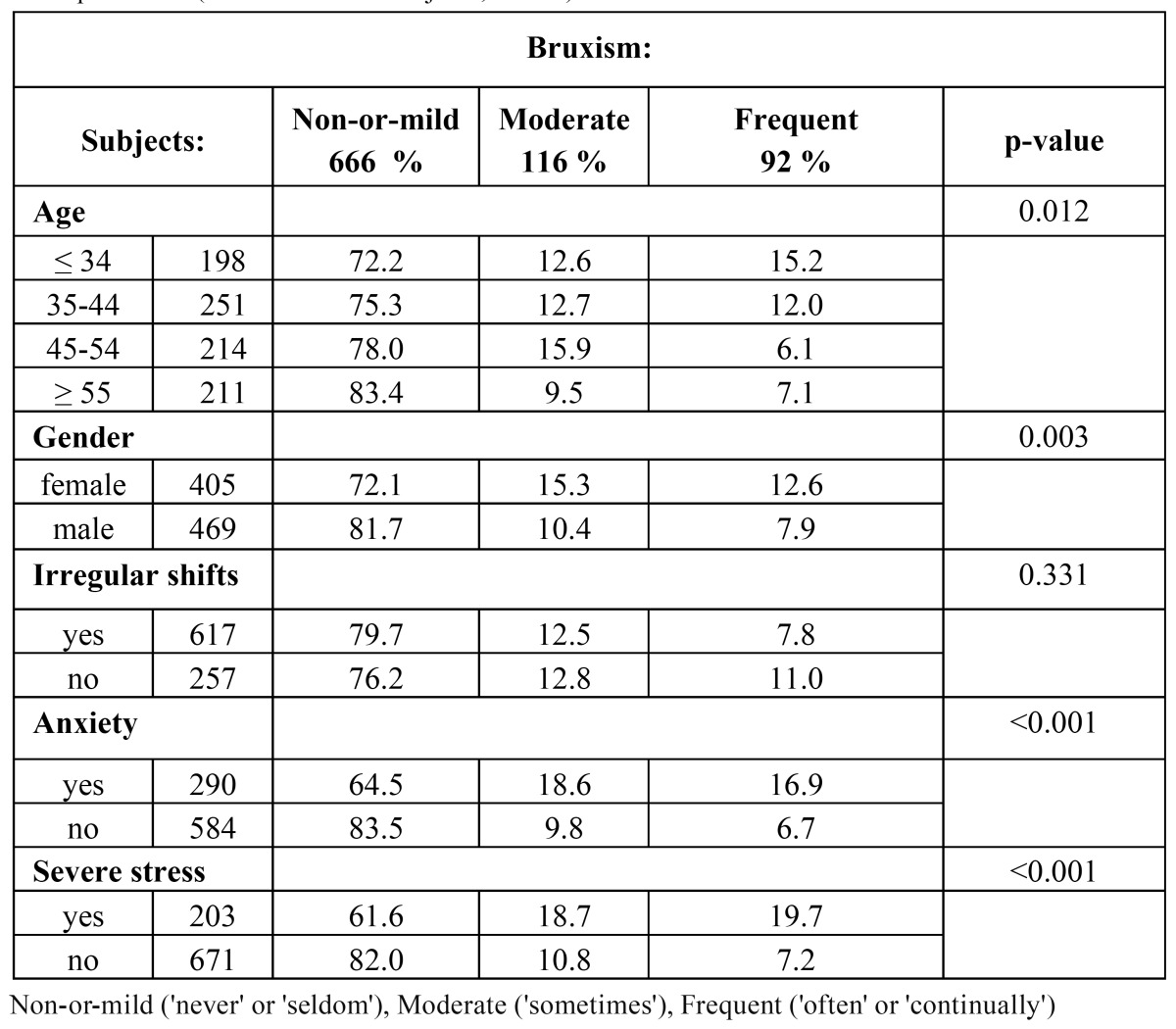
Self-assessed severity of bruxism by age, gender, and the studied psychological variables. Chi square test. (total number of subjects; n=874).

**Figure 1:**
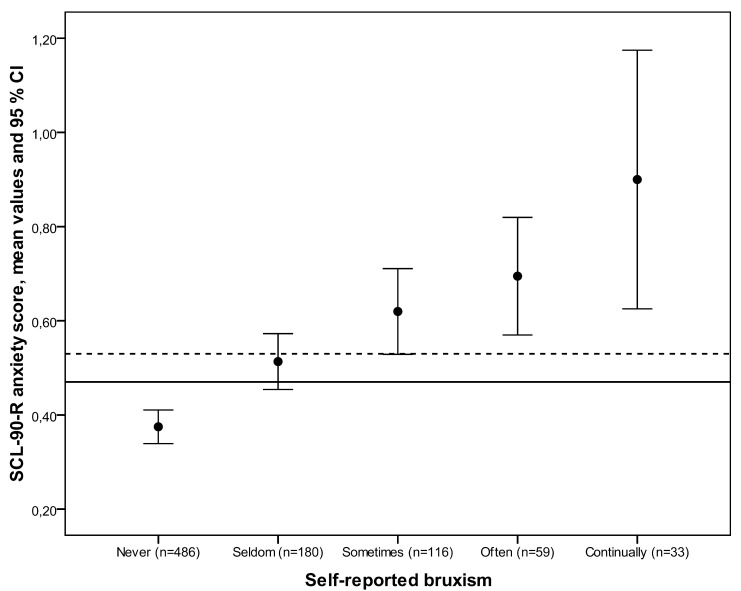
Mean SCL-90-R anxiety raw scores and their 95% confidence intervals (CI) according to self-reported bruxism. Horizontal line represents the overall mean value among the study population; grid line represents the norm for community subjects in Finland (18). Statistical evaluation by Jonckheer-Terpstra -test to evaluate whether the severity of self-reported bruxism and anxiety were correlated (p<0.001) (p-value, n = number of subjects).

The mean anxiety score for the study population was 0.47 (SD 0.42). Subjects aware of more frequent bru-xism tended to report significantly more severe anxiety, and a clear-cut difference was seen in the anxiety scores between severe bruxists and non-or-mild bru-xists (p<0.001). Also, those reporting frequent bruxism had an anxiety score clearly above the Finnish norm for community subjects (Fig. 1).

Multinomial logistic regression revealed that the signi-ficant associations of both anxiety and severe stress with bruxism were consistent ( Table 2). Anxiety above the overall mean score (OR 2.2; 95 % CI 1.3-3.6) and severe stress (OR 2.5; 95 % CI 1.5-4.2) were significantly more probable among frequent bruxers than those reporting non-or-mild bruxism. Similarly, both anxiety (OR 1.7; 95% CI 1.2-3.1) and severe stress (OR 1.7; 95% CI 1.0-3.1) were associated with moderate bruxism.

**Table 2:**
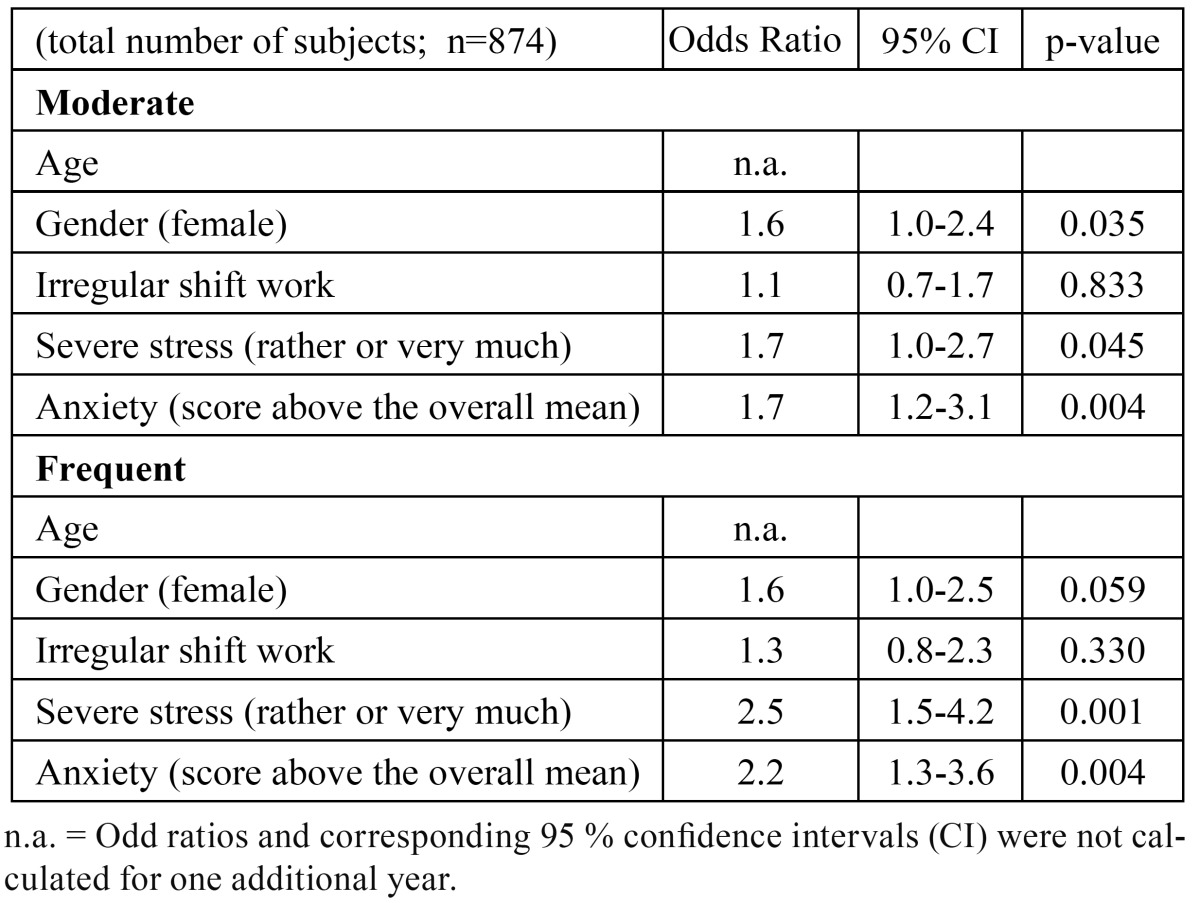
Multinomial logistic regression: independent effects of stress and anxiety on moderate and frequent bruxism. Reference category non-or-mild bruxism. Adjusted by age, gender and work type.

## Discussion

Our main finding was the clear-cut difference between frequent bruxers and non-or-mild bruxers as regards anxiety scores. The present study also confirms previously found associations between self-reported stress and bruxism among non-patients (16,17).

To be useful, rating scales should be reliable (i.e., consistent and repeatable even if performed at different times or under different conditions) and valid (i.e., represent the true state of nature). In the present study, anxiety was measured using the SCL-90-R, which has been reliability and validity tested among adults in Finland (18). The mean anxiety score (0.47, SD 0.42) was close to the SCL-90-R norm (0.53, SD 0.49) for community subjects in Finland (18). Also the used single-item five-scale stress question has been shown to be a valid method to measure stress on group level (19). What self-reported bruxism indicates has remained unclear, however. Thus, in our study, it is noteworthy that subjectively assessed severity of bruxism associated significantly with the outcomes of both these psychological measures.

By definition, bruxism events may occur while asleep or awake, although it seems that these two entities do not share their correlates (20). Awake bruxism mainly consists of tooth clenching, while grinding is more rarely noted. Sleep bruxism is more often tooth grinding with phasic (rhythmic), tonic (sustained) or mixed (both types) jaw muscle contractions (21). Electromyographic recordings definitely reveal masticatory muscle activity, and combined with polysomnography (PSG) and audio-video recordings, sleep bruxism and possible concomitant events can be detected (21). However, in epidemiological studies data on bruxism are usually gathered from questionnaires or interviews. Thus, awareness of bruxism in epidemiological studies (including the pre-sent one) most likely includes phenomena other than rhythmic masticatory muscle activity (RMMA) typical of ‘pure’ sleep bruxism. PSG studies also tend to have strict inclusion criteria, while they focus on sleep bruxism (RMMA) mechanism as well as events concomitant to RMMA during sleep (e.g. snoring, sleep apnea, and gastroesophageal reflux). In such studies, subjects with anxiety or stress, for example, are often excluded as these states may have some confounding. Consequently, it may be impossible to isolate the role of anxiety and stress in concomitant autonomic nervous system response and the genesis of bruxism.

The present findings corroborate the few existing studies on the relationship of reported bruxism and anxiety among adult populations (1-10). With the exception of the only large general population based survey of Ohayon et al. (6), however, these studies were performed on much smaller populations. A recent review concluded that awake clenching may be mainly associated with psychosocial factors and various psychopathological symptoms, while data on the etiology and characteristics of bruxism from sleep laboratory studies do not support the association of psychosocial disorders and polysomnographically diagnosed bruxism (22). The authors also suggest that future research should be designed to better distinguish these two forms of bruxism when investigating underlying psychosocial issues (22).

The present findings suggest that self-reported bruxism and psychological states such as anxiety or stress may be related in working age subjects.
